# On Mr. Collier's Proposed Method of Restoring Patients Poisoned by Narcotics

**Published:** 1822-04

**Authors:** Thos. Dugdale

**Affiliations:** Blackburn, Lancashire


THERAPEIA.

**Art. IX.—*On* Mr. Collier's *proposed Method of restoring Patients poisoned by Narcotics.***

*To the Editor of the London Medical and Physical Journal.*

Sir,

In perusing the last Number of your valuable Journal, I find, communicated in the "Notices to Correspondents," a mode proposed, by Mr. Collier, for effectually rousing the system in cases of poisoning by narcotics, which consists in scattering the *pubes dolichi prurientis* over the body of the patient, and particularly about the head, neck, and arms. My motive for thus intruding on your time, is to inform you that the above treatment is by no means a new discovery, as it has been employed, (generally with decided benefit,) during the last twenty years, by my late preceptor, Mr. Barlow, surgeon of this town, in those cases. On my being examined on that subject, at the Apothecaries' Hall, in 1819, I mentioned the above treatment, which was highly approved of by my examiners. Mr. B. has also found a similar practice useful in cases of intoxication, and of suspended animation. I remain, yours, &c

THOS. DUGDALE.

*Blackburn, Lancashire; March* 11*th*, 1822.

